# Firms navigating through innovation spaces: a conceptualization of how firms search and perceive technological, market and productive opportunities globally

**DOI:** 10.1007/s00191-016-0478-0

**Published:** 2016-10-04

**Authors:** Maureen McKelvey

**Affiliations:** Department of Economy and Society, IIE, Institute for Innovation and Entrepreneurship, University of Gothenburg, Gothenburg, Sweden

**Keywords:** Innovation and Invention, Technology Management, Opportunities, Global R&D Management, Innovation Systems, 031, 032, 033, 038

## Abstract

The main contribution of this paper is a theory-based conceptual framework of innovation spaces, and how firms must navigate through them to innovate. The concept of innovation systems - at the regional, sectoral and national levels - have been highly influential. Previous literature developing the concept of innovation systems has stressed the importance of institutions, networks and knowledge bases at the regional, sectoral and national levels. This paper primarily draws upon an evolutionary and Schumpeterian economics perspective, in the following three senses. The conceptualization of 'innnovation spaces' focuses upon how and why firm search for innovations is influenced the opportunities within certain geographical contexts. This means that the firm create opportunities and can span different context, but they are influence by the context in term of the access, flow and co-evolution of ideas, resources, technology, people and knowledge, which help stimulate business innovation in terms of products, process and services. The paper concludes with an agenda for future research and especially the need to focus on globalization as a process of intensifying linkages across the globe.

## Introduction

An area of important contribution – but also a key conceptual problem - for evolutionary approaches to social science is how and why to link micro-level data with macro-level theorizing. 1 van den Bergh and Gowdy (2003) outline the promises and problems of developing the micro-foundations of macroeconomics, from an evolutionary perspective. They address a range of approaches, from multi-agent models including complexity and hierarchy to group selection as well as the need to recognize heterogeneity of firms in analyzing macro processes. More specifically in this line of reasoning, Dosi et al. (2008:601) stress that four basic notions are: 1) innovation is a key driver of economic growth; 2) the heterogeneity of firms; 3) markets are selection devices that affect decentralized decisions like entry and exit; and 4) the above three Schumpeterian micro-foundations are complementary with a Kuznetsian perspective on industrial dynamics. Within management literature inspired by evolutionary economics and organizational learning, a key concept has been ‘firm search’, often related to the creation of economic opportunities (Laursen 2012). This paper focuses upon how to help explain how and why firms differential abilities to ‘navigate’ through three dimensions of innovation spaces influence their ability to recognize opportunities, and therefore, in longer run, be able to compete and generate aggregate economic growth. This paper is an initial step in developing this understanding. This paper therefore uses elements of existing theoretical and empirical understanding in a new combination, in order to propose a new synthetic conceptualisation of the innovation process in firms as the navigation of a complex space involving different types of opportunities.

Empirically, these topics are increasingly important to both firms and economies, due to the globalization of all types of business activities – from research and development through production, service and customers. Archibugi and Iammarino (2002:99) define globalization as ‘a high (and increasing) degree of interdependency and interrelatedness among different and geographically dispersed actors.” Similarly, the authors provide empirical evidence of globalization, and why this shift matters for invention and innovation. Taking this proposition of globalization seriously also means that firms increasingly have to search and develop opportunities globally. Globalization thus also requires a more complex theoretical understanding of how such interdependencies and interrelatedness affect firms, and the observation that firms struggle with search and opportunities on a global scale prompted this line of research to better conceptualize the processes.

A key concept used in this paper is opportunities. Opportunities are most often discussed in recent years within entrepreneurship literature, although also inherent in Schumpeter’s (1934, 1942) conceptualization of the dynamics of the economy. Within entrepreneurship literature, there has been a debate about whether opportunities are created or discovered (Alvarez and Barney 2007; Alvarez et al. 2013). In the creation theory within entrepreneurship literature, the nature of opportunities means that the actions of the entrepreneurs create opportunities, as compared to the situation where opportunities exist and are simply uncovered. *Ex ante*, the persons who become entrepreneurs may not be significant different from those who do not become entrepreneurs – but the process of having been and becoming entrepreneurs probably does change them, *ex post*. Given that firms develop innovations in relation to the creation of opportunities, their knowledge about the future is limited yet as they act, they also create new knowledge (Holmèn and McKelvey 2013). Accordingly, some work has stressed that the firms’ innovative search occurs in an environment of co-evolution with market, technologies and institutions, as widely acknowledge in the innovation system literature (Nelson 1993; Lundvall et al. 2002) and related literature (McKelvey 1996, 2004; McKelvey and Holmèn 2006).

This paper therefore starts from the viewpoint that opportunities are created over time, which is also in line with an evolutionary epistemology (Buenstorf 2007; McKelvey et al. 201). In order to conceptualize whether, how and why firms do, or do not, move through complex space, it is necessary to start from two insights from evolutionary economics: a) that firms search for opportunities under conditions of selection and b) that the ability to identify and exploit that knowledge is related to firm internal capabilities (see review in Salter and McKelvey 2016). The implication is that firm search is necessary because this ability to innovate – and to create opportunities – is not randomly spread over the population of firms in an industry, but is concentrated in firms with certain capabilities and routines. Moreover, in the existing literature addressing evolutionary economics, there are different approaches, methodologies and theories, which this paper makes no claim to address all of them.

This paper only focuses on three core questions of an evolutionary approach to the development of knowledge and innovations in the economy– namely from where do different types of opportunities emerge? Why do we assume that firm search is necessary for innovation? And how does the innovation system help define the industrial, regional and national context, within which firms search? The answers to these questions are used to develop a conceptualization, which includes the microfoundations of firm search with the macro processes involving technology, markets and institutions.

## From where do different types of opportunities emerge?

The first question is, From where do different types of opportunities arise? This question is explicitly addressed within evolutionary economics, in that capitalism is restless (Nelson 1996; Metcalfe 2002). Evolutionary economics focus upon dynamics and emergent systems as well as the peculiar role of knowledge and the appropriation of value of innovation. Industrial dynamics is driven through innovation in large companies and through entrepreneurship through new companies (Schumpeter 1934, 1942). Recent literature has both examined to what extent that Schumpeter was evolutionary and dynamic in his theorizing, as well as strived to define principles for evolutionary economics (Andersen 2011; Witt 2010; McKelvey and Holmén 2006; Foster and Metcalfe 2001). Metcalfe (2002 and 2008) argues that the restless nature of capitalism has to do with knowledge as the pre-eminent source of variation, but that there is a difference between knowledge and information which helps explain why heterogeneity of firms also matters. Here as well, markets are the primary arenas of selection for micro-processes.

From this literature, a key insight for this paper is thus that the creation of opportunities is a process of knowledge accumulation (Metcalfe 2002; McKelvey et al. 2015), and therefore one must understand firm search in relation to the innovation spaces within which they navigate. Moreover, opportunities are generated endogenously through the dynamics of the economic system and not through exogenous shocks. In other words, the phenomena of innovation and entrepreneurship exist because the actors create different types of opportunities, which involve engaging in decisions and actions under conditions of uncertainty and risk-taking.

Existing literature defines innovative opportunities as “the possibility to realize a potential economic value inherent in a new combination of resources and market needs, emerging from changes in the scientific or technological knowledge base, customer preferences, or the interrelationships between economic actors” (Holmén et al. 2007, p. 37). As such, an innovative opportunity consists of the following three components: a) an economic value for someone; b) mobilization of resources; and c) the ability of the actor pursuing the opportunity to appropriate some of the economic value.

Three types of opportunities can be identified in the literature, and the issue is from where each type of opportunity emerges – which leads to the follow-on question for this paper, namely where should the firm be searching?

The first type that firms monitor is technological opportunities, which arise from scientific and technological knowledge (Scherer 1965; Nelson and Winter 1982; Breschi et al. 2000; Oltra and Flor 2003; Palmberg 2004). Note that there appears to be a relatively uneven distribution of ‘technical opportunities’ at different times and in different technologies and industries (Nelson and Winter 1982; Dosi 1982). Breschi et al. (2000) further develop the ‘technological regime’ proposed by Nelson and Winter (1982). They argue that ‘*Technological opportunities* reflect the likelihood of innovating for any given amount of money invested in search’ (ibid). Differences between industry help explain where the generation of these types of opportunities occur. Breschi et al. (2000: 390–391) state that ‘observed sectoral patterns of innovative activities are related to the nature of the relevant technological regime….defined by the specific combination of technological opportunities, appropriability of innovations, cumulativeness of technical advances and the properties of the knowledge base underlying firms’ innovative activities.’ This research implies that sectoral differences impact the ability to find new opportunities and to appropriate the value to the firm.

Technological knowledge is also related to how technology develops more generally in society, usually though a combination of public and private investment into new knowledge (McKelvey 2014; Archibugi and Filippetti 2015). Technological opportunities are thus created through the development of related areas of knowledge, including a range of relevant scientific and technological knowledge bases, techniques, and instrumentation.^2^ Technologies can be seen as general bodies of human knowledge, which are often created and further developed within specific application areas, such as within firms, industries or other societal contexts of use; one must distinguish the stock of ‘useful human knowledge as well as the continuous addition of new knowledge’ (Moykr 2002).^3^ Literature within the history of science and technology and in economic history suggests that such knowledge development is an on-going process, usually driven forward by specific individuals and acting within specific societal institutions (Rosenberg 1982, 1994). Some technologies develop quickly and general purpose technology, whereas other technologies develop slowly.

Technological knowledge is useful because it helps to solving problems, and so there are evolutionary dynamics in the underlying system of innovation whereby solving one technological problem often opens up new technical opportunities and new technical problems of relevance for developing complex engineering products. Solving one technical problem often focuses the attention of decision-makers on the need to improve related complementary technologies, which are known as ‘focusing devices’ for bottlenecks and as ‘reverse salients’ in a large technical system (Rosenberg 1982; Hughes 1983). These technical problems often require search activities in a variety of technological areas, and these search activities and problem-solving activities involve technological and market learning by diverse actors. Relatedly, Stankiewicz (2002) calls the opening up of new areas of ‘cognitive space’ and ‘design space’ as the exploration of new search space, to develop new technological opportunities. This helps explain why the science and university system are potent drivers of economic change. However, the science and business system need to have interdependencies and be interconnected because there are feedback loops between practical application of knowledge within an industry and the broader scientific and technological knowledge (Kline and Rosenberg 1986).^4^ Biotechnology is one example, where scientific progress has stimulated extensive economic development, regeneration of existing firms, and knowledge intensive entrepreneurship (Audretsch and Feldman 1996; Senker 1999; McKelvey 1996; Faulkner and Senker 1995).

The second type of opportunity that firms must monitor is market opportunities. Entrepreneurship literature originally focused upon market imperfections and arbitrage profits, and therefore had a particular focus on how the entrepreneur could use that knowledge to engage in purposeful action (see discussion in Holmèn et al. 2007). Shane (2000) demonstrated how the individual entrepreneurs’ prior knowledge affected cognition and the interpretation of opportunities. This is one type of the market opportunities of interest here, whereby information asymmetries may exist due to the differences in individual cognition, thereby opening up market opportunities.

More broadly, in the economics of innovation literature, however, a debate was originally formulated about the driving forces for the development of new knowledge, which is useful in the economy. A dichotomy was proposed between ‘technology’ and ‘market’ in a dynamic sense, as one classical distinction is between ‘technology push’ and ‘market pull’ (Mowery and Rosenberg 1991). Technology push means that the investments into research and technology lead to the development of new ideas that can be tested and introduced to the market. The technology ‘pushes’ out new ideas. The notion of market pull means that the inventor or innovating firm comes up with an idea, because they are responding to the perceived wants and preferences of customers. In other words, the market ‘pulls’ out creativity and new ideas. In more recent years, the discussion focuses not on markets in general but on the role that lead users often significantly impact the direction of technology development in an early phase (von Hippel 1988; Franke and von Hippel 2003). As the technology matures and diffuses, new types of user groups may express quite different needs than the early ones, thereby influencing both the technology per se as well as the range of potential future uses.

Innovations require both, and because business innovations must ultimately be sold, recent innovation literature stresses that customers, and relevant knowledge about customers and societal links, needs to be integrated into business innovation theory (Dodgson et al. 2014). New business models is one tool to integrate ideas, technology, markets and so forth into what the firm offers to different types of customers and how the firm appropriates value.

The third type of opportunity that firms must monitor is the productive opportunity. This relates to how the firm should organize its resources internally. Penrose (1959) argued that the firm has the possibility to combine its internal resources in many new ways. The ‘productive opportunity’ limits the number of combinations, especially as perceived by managers in the firm. A key issue involves the managers’ capacity to envision alternative modes of using the resources at hand (Penrose 1959: 31–42, 111). In more recent literature, one can argue that the notion of productive opportunities relates to internal firm issues, and what is known as capabilities. By capabilities, we mean the knowledge, experience and skills that firms have which are appropriate for carrying out specific activities (Richardson 1972). Capability building is seen as a major source of sustained competitive advantages for the firm (Barney 1991). Combinative capability can be defined as the ability to integrate and synthesize internal resources and external learning and deploy them in a competitive environment (Kogut & Zander, 1993). Recent literature includes many contributions about capabilities, dynamic capabilities and capabilities as specifically related to innovation (Helfat and Lieberman 2002; Bingham et al. 2007; Teece 2007; Hine et al. 2013). Helfat and Lieberman (2002:725) distinguish between resources ‘as stocks of available factors that are owned or controlled by the firm, and capabilities as the firm’s capacity to deploy resources for a desired end result’. The literature on dynamics capabilities stresses that the firm must adapt to demands in the external environment, and reconfigure resources (Teece et al. 1997). Firms with high levels of motivation and the capacity to learn should be more open to gaining experience from different situations; this makes them more likely to seize pre-emptive opportunities than more defensive firms which employ static resource-exploitation strategies (Teece et al. 1997). Hine et al. (2013:17) distinguish between different types of capabilities, across the dimensions of predominate resources, routine patterning, learning focus and strategic intent. The three levels of capabilities are ‘higher-order dynamic learning capabilities’ which involve creative ability; ‘lower-order dynamic functional capabilities’ which involve dynamic ability; and ‘first-order ordinary capabilities’ which involve static ability. The latter category involves general resources, rigid routine patterning, exploitative focus of learning tasks and subsistent strategic intent. Search processes involve higher-order and lower-order ones.

In summary, this section has addressed evolutionary literature that helps to answer the question, From where do different types of opportunities emerge? These theories help to conceptualize how and why firms are searching for new opportunities, and especially in relation to technological, market, and productive opportunities. Given the evolutionary epistemology, this paper also follows the conceptualization that the firm involved in innovation processes must also be engaged in, and help to create, these three types of opportunities. An active firm – with high levels of motivation and the capacity to learn – is more likely to search and be able to create opportunities across all these three dimensions. Moreover, the literature specifies that firms can be analyzed as engaging in specific, identifiable processes, specifically to identify economic value; mobilize resources; and appropriate at least some of the economic value.

In terms of the conceptualization of firms navigating innovation spaces, a first constituent element is:

Firm help to seize and create opportunities in their innovation space, over time. The innovation space can be thought of – and analyzed – through three axes. The first axis is of creation of technological opportunities through scientific and technological knowledge. The second axis is the creation of market opportunities related to new market and customer knowledge. The third axis is the creation of productive opportunities, dependent upon using business knowledge of how to operate innovation in the firm locally and globally and leading to a reconfiguration of capabilities.

## Why do we assume that firm search is necessary for innovations?

The second question to address, is what does the literature say about, Why do we assume that firm search is necessary for innovation? Search is core to evolutionary thinking, because, a key notion is that firms are heterogeneous in a way that matters to economic change. Selection takes place at the population level, and therefore differences at the unit level matter for determining which organization disappears, or else continues to exist. If the firm does not search – or does not find opportunities – they are likely to disappear, due to market selection.

In relation to firms search for technical innovations, McKelvey (1996) specifies three key points in a conceptualization based upon evolutionary economic theories. Firstly, there is an assumption of intentionality, and that humans generate novelty and diversity, which are intended to satisfy environment conditions. Secondly, because of intentionality in relation to perceived environmental conditions, firms explore certain ‘search spaces which can be presented as a three-dimensional space, full of hills, valleys and plains’. Because the search for innovation does not occur randomly in time and space, this means that certain parts of search space are more explored than other. Thirdly, there is an expectation of diversity at the micro level of firms, which will in turn impact future performance of firms and also of their ability to search for, and learn about, innovations. The reason is that the firm needs to access ideas and information in order to take advantage of different types of opportunities and to innovate in different geographic contexts and cognitive frameworks. A key insight is also that due to heterogeneity, firms will have explored different parts of the innovation space.

Hence, search is not just a question of identifying ‘the right information’ which exists out there. Focusing on search means that the firm’s internal combinations of routines, resources, capabilities, and learning must be taken into consideration, and will also change over time, in response to internal and external conditions (Nelson 1996; Penrose 1959; Teece et al. 1997; Teece 2007). Theories in innovation management explicates how the internal capabilities of the firm is linked to opportunities arising in the environment, thereby stressing the role of relationships and networks for the firm to search, identify and seize relevant business opportunities (Brusoni et al. 2001; Dodgson et al. 2014). The proposition here is thus that firms will have different capabilities to search for information related to opportunities, as well as different abilities to process and use that information to create new opportunities. Moreover, because the focus here is on search activities related to invention and innovation, this means that the firm is searching under conditions of uncertainty and also in an increasingly globalized context.

From an evolutionary perspective, the argument has been put forth that firms have difficulties in developing innovation relevant to new situations, whether these arise from changes in the industry or in the country. Nelson and Winter (2002:29) call it the competence puzzle:

“How can the same organizations be so impressively competent from one perspective and yet so strikingly "bounded" in their rationality? … In the evolutionary view, the key to the puzzle lies in the contrasting demands of different types of situations. High competence is often achievable where skills and routines can be learned and perfected through practice. For individuals and organizations (not to speak of animals), learning guided by clear short-term feedback can be remarkably powerful, even in addressing complex challenges. But that sort of learning does little to enable sophisticated foresight, logically structured deliberation and/or the improvisation of novel action patterns and situations that demand these are rarely handled well.”

This quote suggests that firm have difficulties dealing with the future – and explicitly states that decisions that must rely upon foresight, logically structured deliberation and novel action patterns. This suggests that search is difficult.

Still, a key aspect of understanding innovation spaces is the role of firms, and explicitly that firms must search for new innovative opportunities, and search for solutions to their technical and market problems (Holmén et al. 2007; Laursen 2012). So, one answer as to why firms must search is related to the types of knowledge involved in invention and innovation as well as the particular role of learning. Existing literature outlines that there is an imperative for firms to learn, develop routines, and access new types of inputs as sources of competitive advantage (Nelson and Winter 1982; Fleming and Sorenson 2001; Rosenkopf and Nerkar 2001; Katila and Ahuja 2002). Another way to understand what firms do is that they need to find new knowledge, which helps them solve different types of problems. According to the problem-solving perspective as developed within the knowledge-based theory of the firm, the key task of the manager is to ‘not how to organize to exploit already developed knowledge or capability, but rather how to organize to efficiently generate knowledge and capability’ (Nickerson and Zenger 2004:617). Firms are recognized as the actors, which play the key role in the development of incremental changes in existing technologies and products along a trajectory (Pavitt 1991; Dosi 1982).

Uncertainty is, by definition, a characteristic of the firm search activities, because novelty is involved in invention and innovation. Business innovation in a broad sense can be defined as ‘novelty of economic value’.^5^ Because innovations require novelty, this implies that the processes leading to innovation will occur under conditions of limited knowledge about the future. Therefore, the decision-making context is uncertain, in the meaning proposed by Knight (1921) of not being able to predict or assess the probability of potential outcomes. Firms face uncertainty rather than risk in that uncertainty cannot be measured nor calculated. In contrast, risk refers to situations where the odds can be predicted, even if we do not predict the outcomes.

But what does it mean that innovation processes involve uncertainty and risk-taking? This paper proposes that: 1) innovation by definition includes an element of novelty for the future, and the novel idea may or may not work; 2) decision-makers are unable to access or process all relevant information; 3) often the future is uncertain in the sense of unknown-able rather than risky in the sense of calculate possible alternatives and 4) multiple possible paths of scientific and technological alternatives can exist to solve any identifiable user needs.

From the firm’s perspective, they are investing in search activities about innovations of which they cannot predict future outcomes. In order to innovate, the firm must make complex decisions under conditions of uncertainty about future technologies and future market preferences. In the Campbell (1960) sense, blind-variation helps explain that the development of knowledge cannot necessarily be predicted, but once new knowledge is developed, there is a change in the later direction of search for new knowledge. In relation to innovations and technical development, these blind-search paths whereby later search is affected should lead to path dependency. In that sense, failures are an inherent part of testing and selection among a variety of alternatives to solve any one problem (Basalla 1988; Vincenti 1990; Dosi 1982, 1988). The literature on radical versus incremental innovations suggests that the degree of uncertainty and the degree of risk may, however, be different in different industries and types of innovations.

In summary, this part of the literature motivates the focus here upon firm search, under conditions of uncertainty about the future as well as making decisions involving risk. An evolutionary perspective of creation of opportunities suggests that the firms (actors) must be understood as heterogeneous actors, with diverse sets of knowledge and capabilities. That is a given, if selection is to occur over time on the population level. Moreover, firms have difficulties in searching for areas further away from their existing resources and capabilities and focus on knowledge that is useful for problem-solving.

In terms of the conceptualization of firms navigating innovation spaces, a second constituent element is:

Firms must navigate through an innovation space, in an evolutionary process, as they search and develop different types of business innovations. By definition, an innovation entails the unknown, as the concept focuses upon novelty of economic value.

## How does the innovation system help define the industrial, regional and national context, within which firms search?

This section addresses the question, How does the innovation system help define the industrial, regional and national context, within which firms search?

Previous literature developing the concept of innovation systems has stressed the importance of institutions, networks and knowledge bases at the regional, sectoral and national levels, known as innovation systems (Malerba 2002, 2009; Edquist 2006; Lundvall et al. 2002; Cooke et al. 1997; Nelson 1993; Edquist and McKelvey 2000). This provides an understanding of the context for innovation processes. Perhaps because economic geographers develop notions of space and proximity, it makes sense to help define the context from their discussions first (Boschma and Frenken 2006; Boschma and Martin 2010; Boschma 2012). In Boschma and Martin (2010: 6–7), an ambitious range of scholarship for an evolutionary economic geography approach is staked out:

The processes by which the economic landscape – the spatial organisation of economic production, circulation, exchange, distribution and consumption - is transformed over time….spatialities of economic novelty (innovations, new firms, new industries, new networks), with how the spatial structure of the economy emerge from the micro-behaviours of economic agents (individuals, firms, organisations); with how, in the absence of central coordination or direction, the economic landscape exhibits self-organisation, and with how the processes of both path creation and path dependence interact to shape geography of economic development and transformation, and why and how such processes may themselves be place dependent.

The current paper shares an intellectual heritage and interest in these types of questions; although the purpose here is much more narrow than their agenda. Still this quote identifies many of the core issues about self-organization, path dependency and microfoundations that are also found in evolutionary economics.

That leaves us with the task of exploring how theoretical insights can be applied to understand the micro-behavior of firms, as agents navigating through complex spatial and industrial dimensions, and in ways this will impact their future development of innovations.

This brings us back to how microfoundations are linked to macro processes. In this conceptualization, it is useful to define an innovation space, because there is a relationship between the development of firm capabilities and the innovation system. The innovation system provides many elements, such as access to networks, institutions, as well as technology and science. Firms do not search randomly, nor does it only occur inside the firm. Innovation processes rely upon search outside the firm, and innovation is distributed in the sense that actors are distributed across countries, industries and different types of firms and organizations (Metcalfe 2008).

**Fig. 1:**
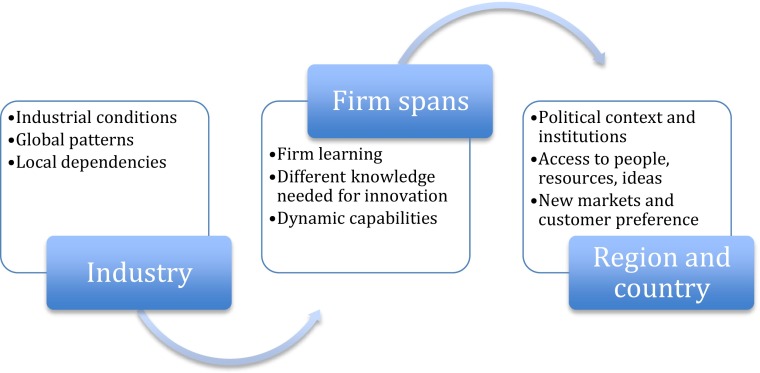
Interactions as the firm spans between industry and region/country

Thus, the proposal here is to draw upon these insights from innovation systems, to express that firm search activities must span industries as well as regions/countries. This is visualized in Fig. 1 below.

The firm is conceptualized as spanning the industry and the region/country level, in the sense that they must search this external environment in order to find relevant new knowledge and create opportunities. The visualization in Fig. 1 suggests that there are interdependencies, or co-evolution, between the firm and contexts in which the firm is active, as explained below.

At the industry level, the innovation space takes it specific form (expression) through a combination of industrial conditions, global patterns, and local dependencies. The industrial sector, or sectoral system of innovation is likely the basis of firm search and learning, rather than the nation (Malerba 2001). The industry life cycle literature claims that rapidly changing technologies lead to the development of new industries (see Abernathy and Utterback 1975; Utterback 1994; Klepper 1996). In the early phase, many competing firms exist, and they compete on alternative product designs; in the later phase, a dominant design emerges; competition is based on economies of scale; and the firms tend to become larger in order to reap scale economies while managing such processes. Hence, the stage in the industry life cycle will also affect the ability and direction of the firm’s search for innovations.

At the region and country level, the innovation space takes it specific form (expression) through a combination of political context, agglomeration effects such as access to people, resources and ideas, and the development of new markets and customer preferences, the national innovation system, including societal structures and institutions. Some literature links this national level of technological and market learning to institutions and social structure, which are simultaneously embedded in the firm, sector and/or region (Fransman 2001; Harvey et al. 2003). Much of economic geography suggests that regions and nations are key geographical spaces, and scales include the regional and national. Therefore, even though this literature is vast and different arguments about the importance of local, regional, national, international levels have been put forward: the concepts of geographical scales or level of analysis are useful here.^6^ This line of research provides the insight that a) geographical contexts can differ in important ways at different scales; and 2) firm networks can help link different innovation spaces to each other through the operations of the firm.

In summary, the firm is said to be searching in space, which links different actors, and the definition of that space consists of innovation systems, where the context may be industrial regional and national, or all three at the same time. From the perspective of the firm, they need to use their networks and organizational structure in order to explore the different opportunities possible in different geographical scales. What is key to the conceptualization here is that firms are the actors, which are spanning between the industry and the regional/national institutional setting. The proposition is: the notion of distributed innovation processes implies that geographic space, context and scale may matter, but that the international knowledge flows as well as firm actions mean that innovation is not restricted to specific places or contexts.

In terms of the conceptualization of firms navigating innovation spaces, a third constituent element is:

An innovation space constitutes a geographical context, which affects innovation through a process of interaction of firms spanning industry as well as the regional/national institutional setting. Because innovation space can be analyzed as this interplay, the innovation space is not bounded by geography per se. Geography matters in that certain regions and countries tend to have a concentration of potential opportunities. However, firms can span across several spaces through networks and through activities in multiple countries and thereby the firm can configure its capabilities to link innovation spaces through the operations and capabilities of the firm.

## Conclusions: a conceptualization and future research agenda

The contribution of this paper is intentionally at a high level of abstraction in conceptualization, given the early stage of this line of research, although a few comments related to research design are included below. This paper has focused on three core questions of an evolutionary approach to the development of knowledge and innovations in the economy– namely from where do different types of opportunities emerge? Why do we assume that firm search is necessary for innovation? And how does the innovation system help define the industrial, regional and national context, within which firms search? The answers to these questions have been used to develop a conceptualization, which includes the microfoundations of firm search with the macro processes involving technology, markets and institutions.

The proposed concept of 'innovation spaces' developed in previous sections – and synthesized below – can thus be understood as a systematic manner in which to organise the various models of the nature and function of what firms do as they search through the space of opportunities, under conditions of uncertainty, seeking to discovery new source of value.

### Conceptualization

Putting together the three constituent elements, the conceptualization of firms navigating innovation spaces, as proposed in this paper is:

Firm help to seize and create opportunities in their innovation space, over time. The innovation space can be thought of – and analyzed – through three axes. The first axis is of creation of technological opportunities through scientific and technological knowledge. The second axis is the creation of market opportunities related to new market and customer knowledge. The third axis is the creation of productive opportunities, dependent upon using business knowledge of how to operate innovation in the firm locally and globally and leading to a reconfiguration of capabilities.

Firms must navigate through an innovation space, in an evolutionary process, as they search and develop different types of business innovations. By definition, an innovation entails the unknown, as the concept focuses upon novelty of economic value.

An innovation space constitutes a geographical context, which affects innovation through a process of interaction of firms spanning industry as well as the regional/national institutional setting. Because innovation space can be analyzed as this interplay, the innovation space is not bounded by geography per se. Geography matters in that certain regions and countries tend to concentrate opportunities. However, firms can span across several spaces through networks and through activities in multiple countries and thereby the firm can configure its capabilities to link innovation spaces through the operations and capabilities of the firm.

The perspective taken here is that of the economist, using tools from an evolutionary analysis of knowledge for invention and innovation, in order to understand why and how the microfoundations matter. The focus is put upon the firm searching and creating opportunities, of which three types have been identified. The three types of opportunities that are created are proposed to entail technological, market, and productive opportunities. Given the focus on invention and innovation, the business managers are making decisions in a dynamic context with Knightian uncertainty. The metaphor of firms navigating innovation spaces suggests the importance of directionality, spatial, and context.

### Future research agenda

Researchers interested in the future research agenda should tackle key theoretical issues, including develop testable hypotheses as well as tackle empirical issues related to research design and especially, how to use data to capture what firms are doing and why it differs.

In order to help structure a future research agenda, Table 1 provides a synthesis of the conceptualization, as divided into the three constituent parts. The left hand column summarizes the main points, when answering the three questions relevant to evolutionary approaches. The middle column states the main theoretical challenges, which should be further developed, including proposing hypothesis. The right column identifies some of the empirical challenges, which are naturally aligned with the theoretical challenges as well

**Table 1:** Conceptualization of firms navigating innovation spaces: Constituent elements, theoretical and empirical challenges

	Theoretical challenges	Empirical challenges
Part I: Opportunities are created in process These represent different types of innovative opportunities	Explain the creation of opportunities Delineate the three types of opportunities – technological, market and productive	Propose how the search for certain types of innovations involve different degrees of uncertainty vs risk Describe firm search and problem-solving in the context of uncertainty and knowledge accumulation
Part II: Firms navigate and search for innovation, under conditions of uncertainty	Discuss uncertainty vs risk Define innovations Describe firm search and problem-solving	Propose how heterogeneous firms can span the context of sectoral, regional and national innovation systems in ways which affect the direction and rate of innovations introduced Explain in detail how the firm can use networks and organizational/operational capabilities to access multiple innovation spaces
Part III: Firms span the industrial, regional and national institutional context	Define context of sectoral, regional and national innovation systems Discuss how the firm can use networks and organizational/operational capabilities to access multiple innovation spaces	Explore how the firm engages in the creation of opportunities while spanning the industry and regional/national context Delineate whether the three types of opportunities – technological, market and productive – are visible to firm managers

In terms of empirical strategy for the research design, the following provides some initial comments about how to tackle the issues. A key scientific challenge is that given the theoretical propositions about the heterogeneity of firms in terms of both capabilities and search, this implies that the research design must take into consideration how the firm interprets its environment, and cannot just assume ‘objective data’ will be processed the same way by all firms.

For the first constituent element in developing a research design to study opportunities, the existing innovation and management literature provides multiple tools that have already been operationalized and validated. In order to study technological opportunities, there are a range of refined indicators of publications and patents as being indicators, respectively, of science and technology as well as many measures of ‘impact’ through citations, referencing, authorship/ownership and so forth. In order to study market opportunities, the data often used includes both quantitative numbers of the size of markets, usually on the vector of increase, as well as more qualitative input on the role of users within innovation processes. In order to study productive opportunities, quantitative tools that relate to internal management control as well as to more qualitative concepts like dynamic capabilities are needed, in a longer time frame. Interesting questions about opportunity creation, for example, is whether, how and why entrepreneurial firms transit from being focused upon technological opportunities primarily, to also take into consideration market and productive opportunities?

For the second constituent element of firms navigating, the research design should take into consideration that innovation spaces are multi-dimensional. Moreover, the word ‘navigating’ indicates the active and heterogeneous nature of firms. Firms may not always be moving and navigating – but search suggests the firms are engaged in exploring new areas and thereby must develop maps and navigate their ways towards new goals. Thus, a detailed empirical strategy combined with an historical methodology could be a useful approach. Search could be made more specific, relative to existing literature, such as existing taxonomies of different kinds of innovations as related to a specification of the degree of uncertainty and/or risk-taking. For example, firm search activities can be mapped in terms of high/low uncertainty and in terms of whether the search pertains to technological, market or productive opportunities. Finally, especially in industries in crises, such as the pharmaceutical industry in recent years, firms at time congregate around the same innovation spaces and use the same search strategies, while at other times, much diversity can be observed. One interesting question is then, How firm who change their strategy to more globalized networks also change their global networks? This could also be done comparative, between firms initially having similar technological knowledge (as signaled by patents and publications for example).

For the third constituent element, developing a research design should draw extensively on concepts and variables found in economic geography and innovation systems. This literature can be used to operationalize and validate the importance of variables such as institutions, networks, flows, etc. For example, literature could further develop the analysis of firms in emerging markets, which try to catch up with developing countries, as a dynamic process of co-evolution, between firms and countries (Malerba and Nelson 2012; Bell and Pavitt 1993). Finally, given that co-evolution over time is expected to occur, then theoretical as well as empirically grounded propositions can be developed. These propositions may help explain different trajectories of development, when firms search in different geographical contexts.

In terms of theoretically interesting issues, there are many to tackle. This paper started by focusing upon the challenges of research based upon microfoundations, and the four basic notions, as outlined by Dosi et al. (2008:601) are: 1) innovation is a key driver of economic growth; 2) the heterogeneity of firms; 3) markets are selection devices that affect decentralized decisions like entry and exit; and 4) the above three Schumpeterian micro-foundations are complementary with a Kuznetsian perspective on industrial dynamics. Each of these notions could be further developed, specifically in relation to innovation spaces and firm search. For example, because search and innovation processes generally occur before, as well as concurrently with, market processes, this means that market selection is not the only selection mechanism. Thus, from an evolutionary and Schumpeterian economics perspective, future research should return to fundamental theoretical issues, such as which mechanisms and processes tend to generate novelty, retain capabilities and attributes, and select amongst alternatives.

Finally, an important topic for future research is to develop the conceptualization, in order to more deeply understand the globalization of invention and innovation. Indeed, this paper was inspired by earlier empirical work to try to understand the rise of Asia – both as economies and as companies located in those countries (McKelvey and Bagchi-Sen 2015). The resulting book examined ongoing process of global interdependencies and interrelatedness, by looking within Asia as well as Asian firms moving to Western countries and at Western firms moving into Asia. In other words, the book provides some empirical evidence and some conceptualization of what Asia means to global invention and innovation. The book argues that four myths are debunked: a) There is a lot of talk about Asia, but it is only talk. Government policy and national institutions are not supportive of technological development and innovation b) Firms from Asia tend not to be entrepreneurial c) Large firms from Asia tend not to be innovative. They focus on low cost, and not on investing in resources to compete through technology and innovation d) Western firms can easily move to outsource customer development, technological development and research and development to Asia. Hence, much more work should be done on issues of globalizaton in general and of Asia in particular, because Asia is quickly becoming a key innovation space where technological, market and productive opportunities are being created. The complexities of globalization of invention and innovation involving firms and countries is an area where theoretical developments are needed, and aligned with empirical studies.

## Footnotes

1 Acknowledgements to the financiers, specifically the Sten A Olsson Foundation for Research and Culture; Getinge; Volvo AB; and SKF. I would also like to acknowledge my co-author Sharmistha Bagchi-Sen, especially our co-edited book on related topics.

2 The rate and distribution of change across technological areas can be measured empirically in many different ways, including studies of technical aspects, such as performance of new materials, as well as studies of more interrelated economic-technological aspects, such as the ratio of price to performance, or second-order effects on demand.

3 Moykr (2002) also proposes a conceptualization of two types of useful knowledge, namely ‘propositional’ (what) and ‘prescriptive’ (how). He argues that much of technology is about ‘prescriptive’ knowledge but is based upon much ‘propositional’ knowledge.

4 The use of techniques and instrumentation as well as the use of machines and capital equipment in factories were initially studied, as places where users impacted technical development.

5 Note that the concept of business innovation as economic phenomena includes a notion of ‘economic value’. Thereby innovation must be linked to a conceptualization of customer and markets, and not just remain at the stage of an idea. That is why the innovation literature generally distinguishes invention – such as an idea or technical item – from innovation – such as a new product introduced to the market.

6 Economic geography and human geography have extensive work on related concepts, including a recent special issue of *Regional Studies* (Boschma 2012; Healy and Morgan 2012; Shearmur 2012).
